# The impact of sleep deprivation and alcohol on driving: a comparative study

**DOI:** 10.1186/s12889-020-09095-5

**Published:** 2020-06-22

**Authors:** Joanna Lowrie, Helen Brownlow

**Affiliations:** 1 University of Dundee, Nethergate, Dundee, DD1 4HN Scotland; 2 The Centre for Forensic & Legal Medicine, 2 Park Pl, Dundee, DD1 4HR Scotland

**Keywords:** Driving, Road traffic accident, Sleep deprivation, Alcohol, Coffee, Caffeine

## Abstract

**Background:**

There is concern about the detrimental effects of shift-workers’ increasing working hours particularly when driving sleep deprived. The approach to measuring the magnitude of driving impairment caused by sleep deprivation was by comparing it to alcohol. The study compared driving performance after 24-h of wakefulness to performance with a BrAC of just over 22 μg/100mls of breath which is equal to 50 mg of alcohol per 100mls of blood (Scottish drink-drive limit). The effectiveness of coffee as a countermeasure for driver fatigue and the association between subjective impairment and actual performance was also investigated.

**Methods:**

A study of 30 participants (11 male and 19 female; mean age 21) was conducted. Subjects were tested under three conditions: fully rested, sleep deprived, and alcohol intoxicated – BrAC mean [SD] 25.95 μg [2.78]. Under each condition, subjects were tested before and after coffee ingestion. This involved driving simulation (Lane Change Task and Reaction Test) and subjective Likert scales (Karolinska Sleepiness Scale and driver impairment scale). Outcome measures included lane tracking adaptive mean deviation, reaction time, and subjective sleepiness and impairment ratings.

**Results:**

Compared to alcohol, sleep deprived mean reaction times were slower (2.86 s vs. 2.34 s) and lateral control of the vehicle was reduced (lane tracking adaptive mean deviation: 0.5 vs. 0.3). Coffee did not produce an improvement when sleep deprived, and instead, performance deteriorated. Females were less impaired following sleep deprivation than males. Following prolonged wakefulness, the correlation between subjective impairment and actual performance was significant.

**Conclusions:**

It was concluded that sleep deprivation has a greater impact on driving performance than a BrAC of 22 μg/100mls of breath, as measured by driving simulation. Coffee is not an effective countermeasure for sleep deprived driving and drivers’ ability to judge this impairment is suggested to be limited.

## Background

Sleep deprivation is recognised to significantly increase the risk of road traffic accidents [1, 2] and therefore poses a serious public health concern. With drink-driving legislation present in many countries worldwide, it is still surprising that there are gaps in legislation relating to driving sleep deprived. Since many countries have different highway rules and legislation, gathering information on these topics can be challenging. Fewer studies have compared the impact of sleep deprivation and alcohol on driving [3–6], unlike the much larger pool of evidence available from studies that have investigated them individually.

Driving is a complex activity requiring several high-order skills where information relating to the constantly changing road environment must be processed quickly and efficiently to enable the driver to respond appropriately [7]. Factors such as alcohol intoxication, drug ingestion and fatigue are all recognised to adversely affect this process [8]. Specifically, fatigue resulting from sleep deprivation has been demonstrated to increase the likelihood of lane drift [3], impair the ability to maintain a constant speed [4, 9] and increase the likelihood of involvement in a collision [10]. In addition to study methods involving driving simulation, two on-the-road studies investigated one night of sleep deprivation on driving performance and convincingly found that lateral control of the vehicle was also severely impaired following sleep deprivation [11, 12]. Furthermore, compared to older drivers, adolescents and younger adults (aged 16–29) are particularly susceptible due to driving inexperience and overconfidence in their driving competence [13, 14].

At each positive BAC level, research indicates an increased risk of a driver being involved in an RTA. The risk rises rapidly and becomes statistically significant after a driver reaches or exceeds a BAC of 0.5 g/L compared to those with zero alcohol in their blood [15]. At a BAC of 0.5 g/L, Mitchell et al. reported impairment of cognitive functioning in drivers including the ability to process information and make decisions under conditions of divided attention [16]. At this level drivers’ judgement of speed and distance will be impaired alongside an increase in risk taking behaviour [17]. Furthermore, drivers are more likely to suffer visual impairment, drive faster than required, have slower reactions to red lights and brake lights, and have an impaired judgement of distances when approaching bends [18].

The optimal times for initiating sleep and wakefulness are determined by circadian rhythm [19]. However, these times are not always suited to the sleep patterns dictated by the economic interests and activities of modern life. Modern sleep patterns which fall outside the natural cycle, such as shift-work, can cause sleep deprivation severe enough to place the individual at an increased risk of accidental injury, particularly when driving, with several studies highlighting healthcare workers as those frequently affected [20–24].

The British Highway Code, therefore, states that a driver must be ‘fit to drive’ and that drivers must minimise the risks of driving when tired, suggesting that the most effective methods of achieving this are by taking a short 15-min nap or consuming a caffeinated beverage before driving [25]. However, while studies into the benefits of taking a short nap have been demonstrated to improve driving performance [14, 26, 27] there have been far fewer investigations conducted specifically into the effects of coffee. Previous studies have demonstrated that dosing with a caffeine supplement of 300 mg can effectively counteract acute sleep deprivation whilst avoiding dangerous cardiovascular and neurological effects [28–30].

The primary aim of this study was to investigate the impact of sleep deprivation on driving ability, using the Scottish drink-drive limit as a benchmark for impairment. The study also sought to investigate whether the consumption of coffee, commonly used as a counter measure to both fatigue and intoxication, produced any beneficial effect (subjective and objective) on driving ability in the sleep deprived.

## Methods

The study had a repeated measure design. The study sample consisted of 30 young adult volunteers (female n = 19, male n = 11) with an average age of 21 years (SD ±1.56). They all possessed valid UK driving licences. We excluded individuals (a) who were taking medication known to affect the sleep/wake cycle, (b) undertaking shift-work, (c) suffering from a sleep disorder, (d) who consumed high level of caffeine (> 5 drinks/day) or (e) with significant previous driving simulator experience. Ethical approval for the study was granted by the University of Dundee School of Medicine Research Ethics Committee and written consent was obtained from participants.

Prior to testing, each participant undertook a training and practice test session in order to familiarise themselves with the driving simulator. Participants then undertook three separate simulated driving sessions (one when fully rested, one after being deprived of sleep for 24 h, and one fully rested but intoxicated by alcohol). Each session was conducted several weeks apart to allow for sufficient sleep pattern recovery. Participants completed a battery of tasks designed to assess their driving ability both before and after consuming a standardised dose of coffee, having abstained from caffeine and alcohol for at least 24 h prior to testing. Coffee dosing was standardised in the form of TASSIMO™ Costa Americano capsules, providing 60-85 mg of caffeine in 220mls.

Sleep deprivation was induced by waking participants at 0700 h and ensuring that they remained awake until the testing session, 24 h later.

Breath alcohol samples were analysed using a Lion Intoxilyzer 9000 machine. Alcohol was administered in the form of white wine (Cambalala Sauvignon Blanc 2017 ABV 12.5%), with doses titrated using the Widmark r-value [31] to produce a target breath alcohol concentration (BrAC) marginally in excess (mean 25.95 μg/100mls, SD 2.78) of the Scottish driving limit of 22 μg/100mls of breath. See Table 1 for individual participant levels. Participants were given 10-min to finish the dose of alcohol. They then had their level checked 30-min post-ingestion (to ensure it was at the desired level) and completed the test battery, before and after coffee.

**Table 1:** Breath alcohol concentrations for all participants

Participant	Mean experimental BrAC (μg/100mls of breath)
1	29
2	27
3	27
4	27.5
5	23.5
6	28
7	27.5
8	25
9	24.5
10	26
11	24
12	23.5
13	24.5
14	27
15	28
16	26.5
17	32
18	22
19	23
20	22.5
21	24
22	25.5
23	23.5
24	22.5
25	31
26	20.5
27	29.5
28	27
29	28
30	29

Driving was simulated utilising a Pro-racing adjustable seat with Logitech Driving Force G920 steering wheel and pedals, configured to produce realistic driver positioning and steering response; a 19″ LG monitor and a JBL Jembe sound system to reproduce road and engine noise, and two driving simulation programmes.

Driving competency was assessed using ‘Michon’s Model of Driving Tasks’ [32]. This model divides vehicular control into three categories: (1) strategic, (2) manoeuvring and (3) operational, with the driver’s ability to make executive decisions [33], their reaction to traffic behaviour, and basic vehicular control being assessed, respectively [7].

### OpenD reaction test (RT)

Assesses driving strategy and manoeuvring ability by examining the ability of participants to promptly react to a stimulus whilst correctly judging whether to brake or change lane [34] using a 5-lane simulated roadway with overhead signage prompts. It generates a test score based on reaction time (milliseconds) and correct decision making in response to speed or lane change prompts.

### 2009 ISO standard lane change task (LCT)

Assesses operational skills by testing steering and lateral vehicle control [35]. Participants, when prompted by signage, were required to perform multiple deliberate lane changes as quickly and efficiently as possible along a 6 km route whilst obeying the indicated speed limit [36]. Driver impairment was quantified by calculating mean deviation from the model lane change path (MDLT) in response to signage alongside the ability to maintain lateral control of the vehicle.

### Karolinska sleepiness scale (KSS)

A 9-point Likert scale requiring participants to select the statement that best describes their current state of sleepiness, where a score of 1 corresponds with feeling extremely alert and 9 with feeling very sleepy and the individual having to make a great effort to fight sleep and remain awake [37, 38].

### Subjective driver impairment scale (SDIS)

A 5-point Likert scale, where 1 indicates the driver considers that they are unable to drive and 5 as being completely capable.

Statistical analyses were conducted using IBM SPSS Statistics version 24 for Windows [39].

## Results

The experimental data was normally distributed but skewed and Friedman’s ANOVA test was used to examine the significance of differences in impairment between test conditions and before and after ingesting coffee. The significance of any differences between select conditions was tested using Wilcoxon Signed Rank test for non-parametric data. Correlation analysis was used to examine the relationship between some variables. For all statistical tests, results with a p-value < 0.05 were considered significant.

**Fig. 1:**
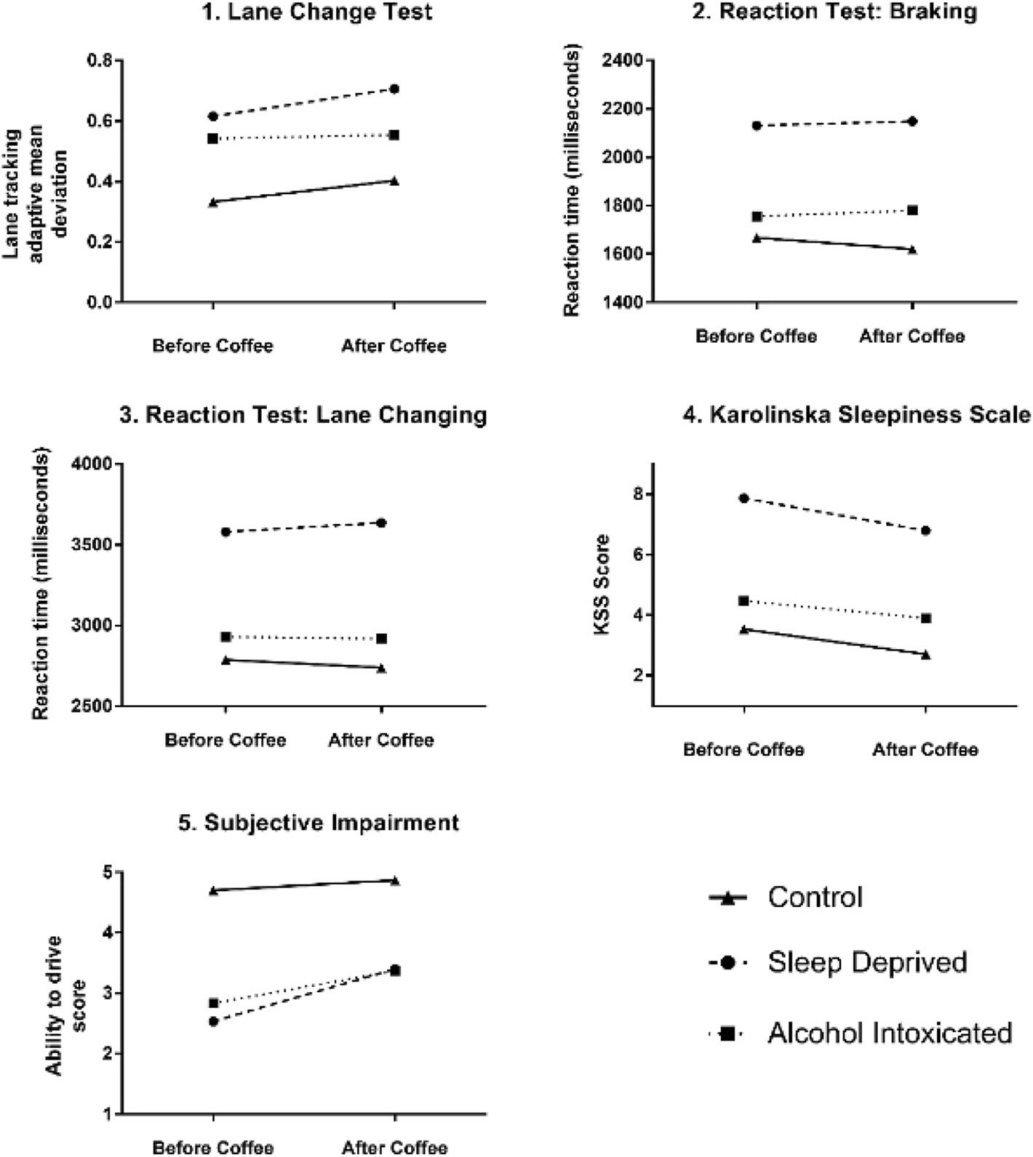
Outcome measure results across all experimental conditions. 1. Lane Tracking Adaptive Mean Deviation (MDLT): Score increases with impairment. 2. Braking Reaction Time (BRT): Score increases with impairment. 3. Lane Change Reaction Time (LCRT): Score increases with impairment. 4. Karolinska Sleepiness Scale (KSS): Score increases with perceived level of sleepiness. 5. Subjective Impairment (SI): Score increases with perceived confidence in driving

Friedman’s non-parametric statistical test of the simulated driving differences across all six repeated measures was significant (P < 0.0001). Figure 1 shows outcome measure results across all experimental conditions.

### Impact of sleep deprivation on driving performance

**Fig. 2:**
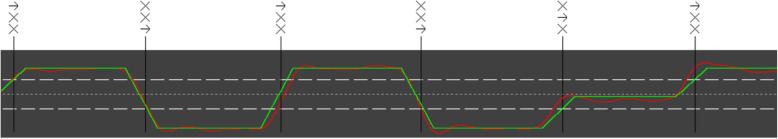
Lane Change Task – extract of Participant 7’s performance when fully rested. The green line illustrates the normative model and the red line illustrates the actual path taken by the driver

**Fig. 3:**
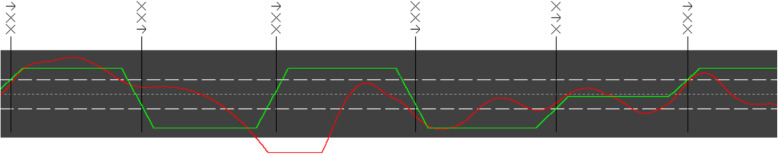
Lane Change Task – extract of Participant 7’s performance following sleep deprivation. Note the slower reaction time response after signage and the larger deviation from the normative path. Just before the 3rd sign the image demonstrates the driver veering off the road after having fallen asleep at the wheel. The greater the difference between the two paths, the greater the driving impairment and thus the higher the MDLT

Sleep deprivation produced a significant detrimental impact on lateral vehicle control (P < 0.0001), braking reaction time (P < 0.0001) and lane change reaction time (P < 0.0001). Cohen’s d effect size for the impact of sleep deprivation = 1.22. Males were affected to a greater degree than females compared to their rested performance (male mean difference in impairment 6.07, female 1.97, P < 0.0071). An example of the detrimental impact of sleep deprivation on Adaptive Mean Deviation of Lane Tracking (MDLT) is illustrated in Figs. 2 and 3.

Although participants reported feeling significantly less impaired (mean SDIS increase of 0.87, P < 0.0001) after drinking coffee when sleep deprived, in reality it was not found to reduce driving impairment (mean combined score 9.03 before coffee vs. 10.16 following coffee; P = 0.01, Cohen’s d = 0.21). Driving performance worsened, with significant deterioration in lane tracking (mean; 0.62 vs 0.71, P = 0.03) and no improvement in braking and lane changing reaction times.

### Impact of alcohol intoxication on driving performance

Alcohol intoxication produced significant deterioration in lateral control (P < 0.0001) and lane changing time (P = 0.0058, Cohen’s d = 1.57) Braking reaction times were also impaired, although not to a significant degree (Table 2). Participants reported feeling sleepier and that their driving was impaired by alcohol, reflected in increases and decreases in KSS and SDIS scores, respectively (Table 2). Coffee did not significantly alter alcohol-related driving impairment across any of the objective domains, although intoxicated participants reported feeling significantly more confident in their driving abilities after consuming it (mean SDIS 2.83 vs. 3.37; P < 0.0001).

**Table 2:** Objective and subjective measures of driving performance

Outcome measure	Rested		Sleep deprived		Intoxicated		P value impact of coffee when sleep deprived	P value sleep deprived vs rested	P value intoxicated vs rested
	Before coffee	After coffee	Before coffee	After coffee	Before coffee	After coffee			
Mean LCT MDLT (SD)	0.33 (0.06)	0.40 (0.09)	0.62 (0.33)	0.71 (0.47)	0.54 (0.17)	0.55 (0.13)	0.03	< 0.0001	< 0.0001
Mean braking reaction time (SD)	1667 (276.5)	1620 (294.5)	2131 (665.9)	2149 (790.2)	1755 (325.5)	1779 (368.5)	0.58	< 0.0001	0.03
Mean lane changing reaction time (SD)	2785 (457.6)	2737 (495.5)	3580 (1371)	3636 (1386)	2928 (566.3)	2917 (557.2)	0.35	< 0.0001	0.0058
Mean KSS (SD)	3.53 (1.55)	2.7 (1.26)	7.87 (0.94)	6.8 (1.35)	4.47 (1.61)	3.9 (1.56)	< 0.0001	< 0.0001	0.02
Mean Ability (SD)	4.7 (0.59)	4.87 (0.35)	2.53 (0.90)	3.4 (0.97)	2.83 (0.83)	3.37 (0.76)	< 0.0001	< 0.0001	< 0.0001

### Comparison of the impact of sleep deprivation with alcohol intoxication on driving performance

**Fig. 4:**
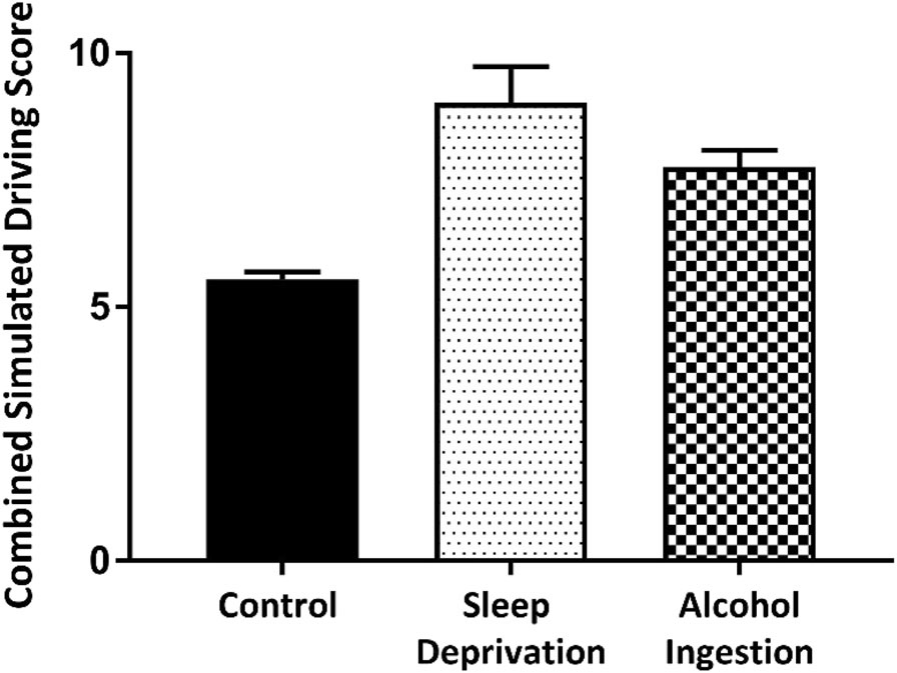
Combined simulated driving scores (RT and LCT) with SEM across the three conditions. (Score increases with driving impairment)

Sleep deprivation was found to produce a greater degree of overall driving impairment than alcohol intoxication when compared to performance when fully rested, with a mean difference of 3.47 (P < 0.0001) in driving score when sleep deprived and 2.22 (P < 0.0001) when intoxicated (Fig. 4). Deterioration in lateral tracking, braking reaction time (P < 0.0001) and lane changing time (P = 0.0003) were all more pronounced when sleep deprived compared to intoxication, with sleep deprivation producing a mean retardation of 0.63 s for combined braking and lane changing reaction times compared to only 0.12 s when intoxicated. Participants reported feeling that their driving performance was equally impaired by sleep deprivation or by alcohol intoxication, with the degree of perceived impairment demonstrating a positive correlation with measured impairment (P < 0.0001). However, following coffee ingestion, sleep deprived participants reported feeling less impaired, despite further deterioration being observed in their driving performance.

## Discussion

### Effects of alcohol intoxication and sleep deprivation on driving performance

Our alcohol intoxication results mirrored the observations of previous studies [40–44], with a demonstrated reduction in lateral control when changing lane whilst intoxicated and a progressive deterioration in the ability to recognise and assess the degree of impairment with an increasing state of intoxication [45]. Additionally, our study also found that this mismatch between real verses perceived impairment was further enhanced following coffee, as participants reported feeling less impaired despite still being in excess of the BrAC limit for driving and exhibiting driving impairment.

Furthermore, the differences in reaction times following sleep deprivation were even more pronounced. Our results support current hypotheses regarding driving whilst sleep deprived. Individual response to sleep deprivation varied widely, with some subjects profoundly affected, as observed through episodes of microsleep and demonstrated through objective and subjective assessment, whilst others were impaired to a lesser degree.

The ability to keep the car centred within the lane is critical to safe driving and a particularly sensitive measure of driving impairment. Our results are consistent with previous research [46], although interestingly, our study found females to be significantly more resilient to impairment, particularly in relation to lateral control when changing lanes. However, whether this difference in impairment between the sexes is due to differences in their sleep behaviour or in their susceptibility to the resultant impairments remains unclear. Braking reaction rate was also detrimentally affected, although to a lesser extent than lane changing rate, which may reflect the more demanding complex decision-making nature of the latter skill, which requires the driver to not only correctly distinguish whether to brake, but also select and manoeuvre into the correct lane.

An explanation for the differences in driving impairment following sleep deprivation and intoxication has yet to be described, but differences in the mechanisms fundamental to both and in strategies employed by subjects to counteract the impairment generated by these two stressors have been proposed [47]. Alternatively, the possibility that both stressors share a common mechanism has also been considered. For instance, intoxication – like fatigue – may enhance the habituation process. This results in the brain becoming progressively susceptible to environmental and circumstantial influences to maintain performance. When these influences are monotonous this habituation might accelerate, “driving” the brain to sleep [48]. However, this is a complex relationship, influenced by several factors such as: demographic differences, emotional states, circumstantial factors and personality traits [49]. Neurologically, one can consider the role of the main inhibitory neurotransmitter in our brain, GABA, as a potential common mechanism. For example, alcohol is well known to be a positive allosteric modulator at GABA receptors and interestingly, Tadavarty et al. found that as little as 12 h of sleep deprivation can alter the expression of GABA_B_ receptors [50].

### Effectiveness of coffee as a countermeasure

The beneficial effects of caffeine on non-sleep deprived subjects are well documented and supported by this study; reaction times improved significantly in the control condition after coffee [51]. However, far from improving, driving impairment when sleep deprived or intoxicated continued to deteriorate despite the consumption of coffee. Factors which may have contributed to this outcome include reduced concentration and fatigue due to the additional time that testing the effects of the countermeasure added to the session, or that the interval between coffee consumption and re-testing was of insufficient duration to allow for the onset of effects. Whilst it has been previously shown that an 80 mg dose of caffeine, delivered in a similar timeframe to our own study produced driving improvement during a prolonged 4-h monotonous simulated drive [52], it may be that coffee has less of a beneficial effect on shorter drives such as commutes or when driving in urban areas with heavy traffic and its associated increased demands in relation to braking and lane changing.

Another possibility is that the caffeine dose was insufficient to produce an observable improvement, as noted in other studies [9, 53]. Many of the experiments that have reported improvements employed significantly larger doses of caffeine than the moderate amounts that would be encountered in real life situations, such as suggested in the Highway Code guidance [25]. 150–250 mg of caffeine (comparable to two/three cups of coffee) has been shown to effectively counteract sleep deprivation (< 5 h spent in bed) when driving in early morning [54] and in the early afternoon [55]. This dose is larger than the one used in this project and the length of sleep deprivation is also shorter. It may be therefore, that coffee improves performance after a shorter length of sleep deprivation than tested in this project.

Two on-the-road driving studies on a motorway in France also suggest differing results – caffeine improved night-time driving in young and middle-aged drivers [56]. Additionally, one study showed that coffee (150-200 mg caffeine) and napping at night significantly reduced driving impairment without altering the subsequent sleep [57]. However, limited comparisons can be drawn from these; on-the road testing has been shown to produce different results from simulated driving. For instance, impaired performance has been noted to occur faster in simulated driving relative to actual driving; most likely as a result of the greater monotony associated with simulated driving [58].

The act of taking short periods of sleep (napping) has been demonstrated to be comparatively more effective in younger subjects, suggesting that the impact of sleep as a countermeasure to sleep deprivation varies with age [56]. The possibility that variance in the effectiveness of coffee as a countermeasure may have occurred, in part with the age of the subject, was eliminated in this study through selecting subjects from a narrow age range. Therefore, the possibility that coffee may be more effective as a countermeasure in an older population cannot be discounted.

### Subjective recognition of impairment

In addition to objectively measuring driving impairment, the study sought to establish the subjects' awareness of an impairment and the impact that coffee had on the confidence they placed in their driving abilities.

Baranski found that actual performance and subjective performance were closely related during periods of prolonged wakefulness [59]. This is consistent with the majority of findings from our study, showing the participants’ perceived level of impairment significantly correlated with their actual driving impairment when sleep deprived and following alcohol. As with other studies [60], participants reported feeling that their driving was impaired and noted a deterioration in their driving performance when sleep deprived compared to when fully rested. A strong correlation was observed between driving impairment and sleep deprived KSS scores for each of the three different tests in all 30 study participants. This is consistent with a study which concluded participants were good at estimating their sleepiness if presented with a task involving some form of objective measure [61]. In contrast to alcohol intoxication, which has been demonstrated to increase a driver’s confidence in driving ability [3], sleep deprived drivers appear to have better insight into the associated impairment of their abilities, as indicated by their subjective driver impairment scale scores. Participants showed a mean 2.16 decrease in subjective impairment when sleep deprived but showed a 1.86 decrease when intoxicated.

We observed that sleep deprivation-associated driving impairment continued to deteriorate following coffee ingestion, despite drivers reporting an enhanced state of alertness and increased confidence in their fitness to drive. This highlights a concerning false perception amongst drivers that coffee will effectively counteract any driving impairment caused by sleep deprivation.

### Limitations

The validity of driving simulator studies will always be challenged because they are limited by how much they replicate real on-the-road driving. Although simulator studies may not completely represent real driving, they have been validated for assessing aspects of performance fundamental to driving, such as steering deviation, reaction time and vigilance [36, 62]. Additionally, the driving scenarios used demonstrate to a significant extent situations from which the project findings can be extrapolated. A simplistic scenario was chosen to allow generalising to motorway driving which is important because drivers experience the greatest risk during monotonous motorway drives [63]. The order of experimental conditions was not randomised or counterbalanced so the study cannot discount the possibility of order affecting the results. Participants were however, given a pilot session to familiarise themselves with the test battery and moreover, the tests themselves were simplistic to eliminate the risk of practice effects. If these had occurred, an improved performance would be expected after each repeated exposure to the test battery. Yet, it can be confidently said that the observed results were minimally influenced by practice because of the pattern they produced. If practice was a factor, if anything, the degree of driving impairment following sleep deprivation was probably reduced. Subjects may have aimed to demonstrate a more substantial impairment after sleep loss than alcohol – participant bias. Although, it is believed the results are valid because the hypothesis was not communicated to participants and they were unaware of how the scoring system worked, helping deter intentional poor performance.

## Conclusions

These findings have important implications for road safety, particularly for those drivers involved in shift-work. The data indicates that sleepiness degrades driving performance, reaction times and the ability to maintain and manoeuvre between lanes. The degree of driving impairment induced by a single night of sleep deprivation was found to exceed that resulting from alcohol intoxication sufficient to produce a breath alcohol concentration (BrAC) of 22 μg/100mls, barely over the legal drink driving limit. If alcohol ingestion (at the legal limit) produces less of an impairment than sleep deprivation, it suggests there is perhaps a need to introduce legislation to protect drivers from feeling compelled to drive when fatigued and other road users from the impact of driving errors arising as a consequence of a fatigued driver. Methods currently being investigated include the pupillographic sleepiness test and a potential roadside blood test which, using blood mRNA transcripts, can accurately predict acute sleep loss.

Road users, especially shift-workers, should be provided with more education concerning the potentially fatal risk of driving sleep deprived, effective countermeasures, sufficient provision of staff sleeping areas/on-call rooms and counselling against the false assumption that consuming coffee can eliminate the effects of sleep deprivation. Therefore, there is possibly a need to update the Highway Code guidance on this subject.

## Data Availability

The datasets used and/or analysed during the current study are available from the corresponding author on reasonable request.

## Abbreviations

BrAC: Breath Alcohol Concentration
KSS: Karolinska Sleepiness Scale
LCT: Lane Change Task
MDLT: Lane Tracking Adaptive Mean Deviation
RT: Reaction Test
SD: Standard Deviation
SDIS: Subjective Driver Impairment Scale
UK: United Kingdom

## References

[1] Department for Transport (2016). Contributory factor, reported accidents by severity, Great Britain.

[2] Soares S, Ferreira S, Couto A. Driving simulator experiments to study drowsiness: a systematic review. Traffic Inj Prev. 2020;21(1):29–37. 10.1080/15389588.2019.1706088 31986057

[3] Fairclough SH, Graham R (1999). Impairment of driving performance caused by sleep deprivation or alcohol: a comparative study. Hum Factors J Hum Factors Ergon Soc..

[4] Arnedt JT, Wilde GJ, Munt PW, MacLean AW (2000). Simulated driving performance following prolonged wakefulness and alcohol consumption: separate and combined contributions to impairment. J Sleep Res.

[5] Williamson AM (2000). Moderate sleep deprivation produces impairments in cognitive and motor performance equivalent to legally prescribed levels of alcohol intoxication. Occup Environ Med.

[6] Powell NB, Schechtman KB, Riley RW, Li K, Troell R, Guilleminault C (2001). The road to danger: the comparative risks of driving while sleepy. Laryngoscope..

[7] Berghaus G, Schnabel E, Madea B (2014). Driving aptitude and fitness to drive. Handbook of forensic medicine.

[8] Lenné MG, Triggs TJ, Redman JR (1998). Interactive effects of sleep deprivation, time of day, and driving experience on a driving task. Sleep..

[9] De Valck E, De Groot E, Cluydts R (2003). Effects of slow-release caffeine and a nap on driving simulator performance after partial sleep deprivation. Percept Mot Skills.

[10] Reyner LA, Horne JA (1998). Evaluation “in-car” countermeasures to sleepiness: cold air and radio. Sleep..

[11] Bosker WM, Kuypers KPC, Conen S, Kauert GF, Toennes SW, Skopp G, et al. MDMA (ecstasy) effects on actual driving performance before and after sleep deprivation, as function of dose and concentration in blood and oral fluid. Psychopharmacology. 2012;222(3):367–76. 10.1007/s00213-011-2497-8 PMC3395348 21952668

[12] Jongen S, Perrier J, Vuurman EF, Ramaekers JG, Vermeeren A. Sensitivity and validity of psychometric tests for assessing driving impairment: effects of sleep deprivation. PLoS One. 2015;10(2):e0e117045. 10.1371/journal.pone.0117045 PMC4323110 25668292

[13] Pack AI, Pack AM, Rodgman E, Cucchiara A, Dinges DF, Schwab CW (1995). Characteristics of crashes attributed to the driver having fallen asleep. Accid Anal Prev.

[14] Horne JA, Reyner LA (1995). Sleep related vehicle accidents. Br Med J.

[15] Blomberg RD, Peck RC, Moskowitz H, Burns M, Fiorentino D (2005). Crash Risk of Alcohol Involved Driving: A Case Control Study. DOT- NHTSA.

[16] Mitchell MC. Alcohol-induced impairment of central nervous system function: behavioral skills involved in driving. J Stud Alcohol Suppl. 1985:109–16 http://www.ncbi.nlm.nih.gov/pubmed/3862850. Accessed Jan 2017. 10.15288/jsas.1985.s10.109 3862850

[17] Jones A (2014). Effects of alcohol on fitness to drive. Handbook of forensic medicine.

[18] National Highway Transportation Safety Administration. Blood Alcohol Concentration: BAC Facts. http://www.preventionlane.org/abcs-bac. Accessed 12 Jan 2018.

[19] Kumar V (2017). Biological timekeeping: clocks, rhythms and behaviour.

[20] Richardson GS, Miner JD, Czeisler CA (1989). Impaired driving performance in shiftworkers: the role of the circadian system in a multifactorial model. Alcohol Drugs Driv.

[21] Maycock G (1997). Sleepiness and driving: The experience of U.K. car drivers. Accid Anal Prev.

[22] Ware JC, Risser MR, Manser T, Karlson KH (2006). Medical resident driving simulator performance following a night on call. Behav Sleep Med.

[23] Barger LK, Cade BE, Ayas NT, Cronin JW, Rosner B, Speizer FE (2005). Extended work shifts and the risk of motor vehicle crashes among interns. N Engl J Med.

[24] Knott M, Classen S, Krasniuk S, Tippett M, Alvarez L. Insufficient sleep and fitness to drive in shift workers: a systematic literature review. Accid Anal Prev. 2020;134:105234. 10.1016/j.aap.2019.07.010 31443915

[25] Department for Transport. General rules, techniques and advice for all drivers and riders (103 to 158) - The Highway Code - Guidance - GOV.UK. In: The Highway Code. 2018. https://www.gov.uk/guidance/the-highway-code/general-rules-techniques-and-advice-for-all-drivers-and-riders-103-to-158. Accessed 7 Dec 2017.

[26] Connor J, Norton R, Ameratunga S, Robinson E, Civil I, Dunn R (2002). Driver sleepiness and risk of serious injury to car occupants: population based case control study. BMJ..

[27] Takahashi M, Arito H (2000). Maintenance of alertness and performance by a brief nap after lunch under prior sleep deficit. Sleep..

[28] Cook CJ, Crewther BT, Kilduff LP, Drawer S, Gaviglio CM (2011). Skill execution and sleep deprivation: effects of acute caffeine or creatine supplementation - a randomized placebo-controlled trial. J Int Soc Sports Nutr.

[29] Smith A, Rusted J, Savory M, Eaton-Williams P, Hall S (1991). The effects of caffeine, impulsivity and time of day on performance, mood and cardiovascular function. J Psychopharmacol.

[30] Lagarde D, Batéjat D, Sicard B, Trocherie S, Chassard D, Enslen M (2000). Slow-release caffeine: a new response to the effects of a limited sleep deprivation. Sleep..

[31] Forrest ARW (1986). The estimation of Widmark’s factor. J Forensic Sci Soc.

[32] Michon J. A critical view of driver behavior models: what do we know, what should we do? Hum Behav Traffic Saf. 1985:485–520. 10.1007/978-1-4613-2173-6.

[33] Dewar RE, Olson PLAG (2007). Human factors in traffic safety. 2nd edition.

[34] Math R, Mahr A, Moniri MM, Müller C (2012). OpenDS: A new open-source driving simulator for research. Adjun Proc 4th Int Conf Automot User Interfaces Interact Veh Appilcations.

[35] ISO. Road vehicles — Ergonomic aspects of transport information and control systems — Specifications and test procedures for in-vehicle visual presentation. 2009;2009. ISO 15008/2009.

[36] Young KL, Lenné MG, Williamson AR (2011). Sensitivity of the lane change test as a measure of in-vehicle system demand. Appl Ergon.

[37] Kaida K, Takahashi M, Åkerstedt T, Nakata A, Otsuka Y, Haratani T (2006). Validation of the Karolinska sleepiness scale against performance and EEG variables. Clin Neurophysiol.

[38] Åkerstedt T, Gillberg M (1990). Subjective and objective sleepiness in the active individual. Int J Neurosci.

[39] IBM Corp (2016). Released. IBM SPSS Statistics for Windows, Version 24.0.

[40] Huntley MS, Centybear TM (1974). Alcohol, sleep deprivation, and driving speed effects upon control use during driving. Hum Factors J Hum Factors Ergon Soc..

[41] Roehrs T, Beare D, Zorick F, Roth T (1994). Sleepiness and ethanol effects on simulated driving. Alcohol Clin Exp Res.

[42] Dott AB, McKelvey RK (1977). Influence of ethyl alcohol in moderate levels on the ability to steer a Fixed-Base shadowgraph driving simulator. Hum Factors J Hum Factors Ergon Soc.

[43] Ranney TA, VJG (1986). Task demand and alcohol effects on simulated driving performance. Proc Hum Factors Ergon Soc Annu Meet.

[44] Gawron VJ, Ranney TA (1988). The effects of alcohol dosing on driving performance on a closed course and in a driving simulator. Ergonomics..

[45] Martin CS, Rose RJ, Obremski KM (1991). Estimation of blood alcohol concentrations in Young male drinkers. Alcohol Clin Exp Res.

[46] Park J. C.; May J. F. GD. W. The effects of sleep deprivation on simulator driving as compared with other psychomotor tests. Eff sleep deprivation simulator Driv as Comp with other psychomotor tests. 2007. https://ir.uiowa.edu/cgi/viewcontent.cgi?article=1246&context=drivingassessment.

[47] Hack MA, Choi SJ, Vijayapalan P, Davies RJO, Stradling JR. Comparison of the effects of sleep deprivation, alcohol and obstructive sleep apnoea (OSA) on simulated steering performance. Respir Med. 2001;95(7):594–601. 10.1053/rmed.2001.1109 11453317

[48] Dinges DF, Kribbs NB (1991). Performing while sleepy: effects of experimentally-induced sleepiness. Sleep, Sleepiness and Performance.

[49] McMillen DL, Adams MS, Wells-Parker E, Pang MG, Anderson BJ (1992). Personality traits and behaviors of alcohol-impaired drivers: a comparison of first and multiple offenders. Addict Behav.

[50] Tadavarty R, Rajput PS, Wong JM, Kumar U, Sastry BR (2011). Sleep-deprivation induces changes in GABAB and mGlu receptor expression and has consequences for synaptic long-term depression. PLoS One.

[51] Research. I of M (US) C on MN. 3, Efficacy of Caffeine. In: Caffeine for the Sustainment of Mental Task Performance: Formulations for Military Operations. Washington, DC: National Academies Press (US); 2001. https://www.ncbi.nlm.nih.gov/books/NBK223791/. Accessed Jan 2017. 25057583

[52] Mets MAJ, Baas D, Van Boven I, Olivier B, Verster JC (2012). Effects of coffee on driving performance during prolonged simulated highway driving. Psychopharmacol (Berl).

[53] Beaumont M, Batejat D, Pierard C, Coste O, Doireau P, Van Beers P (2001). Slow release caffeine and prolonged (64-h) continuous wakefulness: effects on vigilance and cognitive performance. J Sleep Res.

[54] Reyner LA, Horne JA (2000). Early morning driver sleepiness: effectiveness of 200 mg caffeine. Psychophysiology..

[55] Horne JA, Reyner LA (1996). Counteracting driver sleepiness: effects of napping, caffeine, and placebo. Psychophysiology..

[56] Sagaspe P, Taillard J, Chaumet G, Moore N, Bioulac B, Philip P (2007). Aging and nocturnal driving: better with coffee or a nap? A randomized study. Sleep..

[57] Philip P, Taillard J, Moore N, Delord S, Valtat C, Sagaspe P (2006). The effects of coffee and napping on nighttime highway driving: a randomized trial. Ann Intern Med.

[58] Lenné MG, Triggs TJ, Redman JR (1997). Time of day variations in driving performance. Accid Anal Prev.

[59] Baranski J. Sleep loss and the ability to self-monitor cognitive performance. Cogn Fatigue Multidiscip Perspect Curr Res Futur Appl Decad Behav Conf. 2011:67–82. 10.1037/12343-003.

[60] Biggs SN, Smith A, Dorrian J, Reid K, Dawson D, van den Heuvel C (2007). Perception of simulated driving performance after sleep restriction and caffeine. J Psychosom Res.

[61] Horne JA, Burley CV. We know when we are sleepy: subjective versus objective measurements of moderate sleepiness in healthy adults. Biol Psychol. 2010;83(3):266–8. 10.1016/j.biopsycho.2009.12.011 20064579

[62] Mattes S (2003). The lane-change-task as a tool for driver distraction evaluation. Quality of Work and Products in Enterprises of the Future.

[63] Thiffault P, Bergeron J (2003). Monotony of road environment and driver fatigue: a simulator study. Accid Anal Prev..

